# Conserved Amino Acid Moieties of *Candidatus* Desulforudis audaxviator MazF Determine Ribonuclease Activity and Specificity

**DOI:** 10.3389/fmicb.2021.748619

**Published:** 2021-11-11

**Authors:** Hiroko Tamiya-Ishitsuka, Masako Tsuruga, Naohiro Noda, Akiko Yokota

**Affiliations:** Biomedical Research Institute, National Institute of Advanced Industrial Science and Technology (AIST), Tsukuba, Japan

**Keywords:** *Candidatus* Desulforudis audaxviator, cell-free expression, sequence-specific ribonuclease, toxin-antitoxin system, RNA binding

## Abstract

The toxin-antitoxin (TA) system, inherent to various prokaryotes, plays a critical role in survival and adaptation to diverse environmental stresses. The toxin MazF, belonging to the type II TA system, functions as a sequence-specific ribonuclease that recognizes 3 to 7 bases. In recent studies, crystallographic analysis of MazFs from several species have suggested the presence of amino acid sites important for MazF substrate RNA binding and for its catalytic activity. Herein, we characterized MazF obtained from *Candidatus* Desulforudis audaxviator (MazF-Da) and identified the amino acid residues necessary for its catalytic function. MazF-Da, expressed using a cell-free protein synthesis system, is a six-base-recognition-specific ribonuclease that preferentially cleaves UACAAA sequences and weakly cleaves UACGAA and UACUAA sequences. We found that MazF-Da exhibited the highest activity at around 60°C. Analysis using mutants with a single mutation at an amino acid residue site that is well conserved across various MazF toxins showed that G18, E20, R25, and P26 were important for the ribonuclease activity of MazF-Da. The recognition sequence of the N36A mutant differed from that of the wild type. This mutant cleaved UACAAG sequences in addition to UACAAA sequences, but did not cleave UACGAA or UACUAA sequences, suggesting that Asn36 affects the loosening and narrowing of MazF-Da cleavage sequence recognition. Our study posits UACAAA as the recognition sequence of MazF-Da and provides insight into the amino acid sites that are key to its unique enzymatic properties.

## Introduction

Highly conserved toxin-antitoxin (TA) systems are found on microbial plasmids and chromosomes and are specific to prokaryotes (Yamaguchi et al., 2011). Microorganisms harboring this system can control their growth and survival under various environmental conditions, such as starvation, oxidative stress, pathogen infection, and heat stress (Fraikin et al., 2020). TA systems are classified into six types (types I–VI) based on the nature and function of toxin and antitoxin molecules (Unterholzner et al., 2013; Lee and Lee, 2016; Lobato-Márquez et al., 2016). In the type II TA systems, the toxin molecule is a stable protein and the antitoxin is a small unstable protein. The type II TA system MazEF is one of the most extensively studied TA modules. The toxin MazF functions as a ribosome-independent single-stranded RNA endoribonuclease, while the cognate antitoxin MazE suppresses MazF activity (Engelberg-Kulka et al., 2005). Under normal conditions, MazE binds to MazF to neutralize its ribonuclease activity (Zhang et al., 2003a). Further, both the MazE -MazF complex and MazE can regulate MazEF transcription by binding to its TA promoter (Zhang et al., 2003a). However, under stress conditions, MazE is degraded by the intracellular protease, thereby releasing MazF (Aizenman et al., 1996; Muthuramalingam et al., 2016). Free MazF specifically cleaves target sequences within mRNAs, tRNAs, and rRNAs (Zhang et al., 2003b; Schifano et al., 2013, 2014, 2016). Furthermore, binding of MazE -MazF complex or MazE to the MazEF promoter is lost, which relieves transcriptional repression (Zhang et al., 2003a).

*Escherichia coli* MazF (MazF-Ec), consisting of 111 amino acids, was first reported to specifically cleave intracellular single-stranded mRNAs with ACA sequences (Aizenman et al., 1996; Zhang et al., 2003b). Since then, the recognition sequences of various MazF homologs have been characterized (Zhang et al., 2005; Zhu et al., 2009; Park et al., 2011; Rothenbacher et al., 2012; Schuster et al., 2013; Miyamoto et al., 2016a, 2017). *Haloquadratum walsbyi,* isolated from a hypersaline pool in the Sinai Peninsula, possesses a unique MazF that recognizes the longest known recognition sequence, consisting of seven bases (UUACUCA) (Yamaguchi et al., 2012). More recently, the MazF homologs of *Methanohalobium evestigatum* and the *Nitrospira* strain ND1 have been shown to recognize not just one but multiple sequences (Ishida et al., 2019; Aoi et al., 2020). These studies show that MazF toxins, which are conserved in various species, are unique ribonucleases with different recognition sequences and lengths.

Analysis of the crystal structure of *E. coli* MazEF (MazEF-Ec) has shown that the MazF-Ec dimer binds to the C-terminus of MazE-Ec, and the (MazE)-(MazF)_2_ complex binds to the N-terminus of MazE-Ec, resulting in the linear heterohexamer (MazF)_2_-(MazE)_2_-(MazF)_2_ (Kamada et al., 2003). Analysis of the crystal structure of *Bacillus subtilis* MazF (MazF-Bs) in complex with the uncleavable UUdUACAUAA substrate RNA revealed the amino acid residues that were involved in MazF-Bs substrate RNA binding (Simanshu et al., 2013). Furthermore, the amino acids at the binding site of MazF-Bs were mutated, and toxicity to *E. coli* transformed with plasmids harboring these mutants was assessed, to reveal the key amino acid residues required for MazF toxicity *in vivo* (Simanshu et al., 2013). In *Mycobacterium tuberculosis* MazF (MazF-mt6), amino acids near the active site have been identified using crystal structure analysis, and their roles in cleavage activity have been investigated using amino acid mutants (Hoffer et al., 2017).

The *Candidatus* Desulforudis audaxviator strain MP104 was found 2.8km below the surface of a gold mine in South Africa, in fracked water at about 60°C with low biodiversity and oxygen levels (Chivian et al., 2008). Surprisingly, this bacterium comprised >99.9% of the microorganisms inhabiting the fracked water (Chivian et al., 2008). Genome sequencing revealed that the *D. audaxviator* genome comprises all genes encoding life-sustaining processes, such as energy metabolism, carbon fixation, and nitrogen fixation (Chivian et al., 2008). More recently, the *D. audaxviator* strain BYF was isolated from an aquifer 2km underground in Western Siberia (Karnachuk et al., 2019). Successful isolation and pure culture revealed that elemental iron is essential for the growth of this strain (Karnachuk et al., 2019). Comparison between the genomes of the South African and Western Siberian strains showed that the only differences between the strains were related to the mobile elements and prophage insertions (Karnachuk et al., 2019). Though data in the Toxin-Antitoxin Database show that the *D. audaxviator* MP104 genome comprises MazEF homologs (Makarova et al., 2009; Xie et al., 2018), the catalytic function of MazF-Daud1831 (MazF-Da) isolated from *D. audaxviator* remains unclear.

In this study, we analyzed the ribonuclease activity and cleavage sequence of *D. audaxviator* MazF obtained using a cell-free protein synthesis system. We were thus able to predict the physiological role of MazF-Da, whose function has remained an enigma. We also analyzed the changes in enzyme activity using mutants of the amino acids highly conserved between several MazF toxins. This allowed us to assess the amino acid sites critical for MazF enzyme activity and recognition sequence determination. Our findings may be useful for predicting these properties of MazF homologs from other microorganisms.

## Materials and Methods

### Plasmids, Synthetic RNA, Primers, and Fluorescent Probes

The pET-24a(+) expression vector harboring *mazE-Da* or *mazF-Da* was purchased from GenScript Japan (Tokyo, Japan). The genes inserted in the two vectors were designed for optimal codon usage in *E. coli* (Supplementary Table S1). All PCR primers used in this study were purchased from Tsukuba Oligo Service (Ibaraki, Japan). Synthetic RNA constructs were prepared as described previously (Miyamoto et al., 2016a). All fluorescently labeled oligonucleotides (Supplementary Table S2) were purchased from Japan Bio Services (Saitama, Japan).

### Site-Directed Mutagenesis and Generation of PCR Fragments for *in vitro* Assays

Single amino acid mutations (G6A, G6D, P15A, P15D, G18A, E20A, R25A, P26A, P26D, N36A, and R85A) were introduced in the open reading frame of the pET-24a(+)-*mazF-Da* plasmid using the PrimeSTAR^®^ mutagenesis basal kit (Takara, Shiga, Japan) and the respective mutagenic oligonucleotide primers (Supplementary Table S3). Next, the pET24a(+)-*mazF-Da* plasmid with the mutated sequence was transformed in *E. coli* strain DH5α cells (BioDynamics Laboratory, Tokyo, Japan) and cultured, and the plasmid was extracted. PCR was conducted using the prepared mutant plasmids or the wild type plasmid as templates to amplify the region from the T7 promoter to the stop codon to obtain linear template DNA for protein expression analysis, which was purified using the QIAquick PCR purification kit (Qiagen, Venlo, Netherlands). Finally, the PCR fragment sequences of all mutants were confirmed using Sanger sequencing.

### Expression Using a Cell-Free Protein Synthesis System and Purification of Wild-Type MazF-Da or MazF-Da Point Mutants

A cell-free protein synthesis system (PUREfrex^®^2.0; GeneFrontier, Chiba, Japan) was used to produce wild-type MazF-Da and its mutants. The amplified linear DNA fragments (1ng/μl) encoding these MazF-Da proteins were incubated with reaction solutions (solutions I, II, and III in PUREfrex^®^2.0) at 37°C for 5h. Protein purification was performed using a Capturem^™^ His tag purification miniprep kit (TaKaRa). The molecular weight and purity of the protein were confirmed *via* SDS-PAGE. Protein concentration was determined using a Bio-Rad protein assay kit (Bio-Rad, Hercules, CA, United States).

### Expression Using an *E. coli* Protein Expression System and Purification of MazE-Da

After transformation of *E. coli* strain BL21(DE3) (BioDynamics Laboratory) using pET-24a(+)-*mazE-Da*, the cells were seeded on Luria Bertani (LB) plates containing 50μg/ml kanamycin and incubated overnight at 37°C. After growth, single colonies were picked, suspended in 10ml of LB medium containing 20μg/ml kanamycin and pre-cultured overnight at 37°C. The pre-culture medium was inoculated in 1l of LB medium. Isopropyl β-d-1-thiogalactopyranoside (IPTG) was added to a final concentration of 1mM when the OD_600_ reached approximately 0.6. Next, the culture was incubated at 37°C for approximately 5h, and the cells were collected *via* centrifugation at 9200×*g* and stored at −80°C. The frozen cells were thawed on ice and mixed well with 20ml of binding buffer (20mM sodium phosphate buffer [pH 8.0], 300mM NaCl, 5mM β-mercaptoethanol, and 50mM imidazole). The cells suspended in the binding buffer were lysed *via* sonication for 15min using Handy Sonic UR-20P (Tomy Seiko, Tokyo, Japan), and the supernatant was collected *via* centrifugation at 4400×*g* for 15min. The supernatant was then filtered through a 0.45μm filter membrane (Millex, Darmstadt, Germany) to remove cellular debris. Next, the supernatant containing His-tagged MazE-Da was applied to a 1ml His-Trap FF crude column (GE Healthcare, Little Chalfont, United Kingdom) and trapped on the column using AKTA pure 25 (GE Healthcare). The column was then washed with binding buffer. Finally, MazE-Da was eluted using an elution buffer (20mM sodium phosphate elution buffer [pH 8.0], 300mM NaCl, 500mM imidazole, and 5mM 2-mercaptoethanol). The molecular weight and purity of the MazE-Da protein were confirmed *via* SDS-PAGE. Protein concentration was determined using a Bio-Rad protein assay kit (Bio-Rad).

### Assessment of the Enzyme Activity of MazF-Da at Various Temperatures

One unit of MazF-Ec (TaKaRa) or 7.1pmol of MazF-Da, and MazF reaction buffer (20mM Tris–HCl [pH 8.0], 1mM dithiothreitol, 0.01% Triton X-100, and 4U of recombinant RNase inhibitor [TaKaRa]) were mixed, adjusted to a total volume of 30μl, and incubated at 4, 37, 50, 60, 70, 80, and 90°C for 10min. The reaction mixture was placed on ice, followed by the addition of substrate RNA 1500-1 (300ng) and incubation at each temperature for 60min. The resultant digested RNA fragments were purified using RNA Clean and Concentrator^™^-5 (Zymo Research, Irvine, CA, United States). Gel loading buffer II (Ambion, Austin, TX, United States) was added to each sample followed by incubation at 95°C for 5min. The purified RNA was separated on a 7.5% polyacrylamide gel containing 7M urea, stained with SYBR Gold (Life Technologies, Carlsbad, CA, United States), and detected using a Typhoon 9210 imager (GE Healthcare).

### Assessment of the Neutralization of MazF-Da by MazE-Da

To test whether MazE-Da neutralized MazF-Da toxicity, 1 or 10pmol MazF-Da was pre-incubated with 20 or 40pmol of MazE-Da in a reaction buffer at 25°C for 10min. Next, RNA 1500-1 (200ng) was added to this mixture followed by incubation at 60°C for 90min. These RNAs were then purified using RNA Clean and Concentrator^™^-5 (Zymo Research). Gel loading buffer II (Ambion) was added to each sample followed by incubation at 95°C for 5min. Samples were separated on a 7.5% polyacrylamide gel containing 7M urea, stained with SYBR Gold (Life Technologies), and detected using a Typhoon 9210 imager (GE Healthcare).

### Identification of the Cleavage Sequence of Wild-Type and Mutant MazF-Da

The cleavage sequence was identified using the protocols described in our previous study (Miyamoto et al., 2016a). Briefly, 0.625pmol of eight RNA mixtures (500-2: 533nt, 1000-1: 1033nt, 1000-2: 1033nt, 1000-3: 1033nt, 1000-4: 1033nt, 1000-5: 1033nt, 1500-1: 1533nt, and 2000-1: 2033nt) was incubated with MazF-Da (100ng) or its mutants (100ng) at 60°C for 90min in a MazF reaction buffer. Phosphorylation, barcode ligation, and sequencing library construction were performed as described previously (Miyamoto et al., 2016a). Massively parallel sequencing was performed using the MiSeq platform with the MiSeq 500cycle reagent kit v2 (Illumina, San Diego, CA, United States) according to the manufacturer’s protocol. Sequence data were analyzed using CLC Genomics Workbench 11 version 11.0.1 (CLC bio, Aarhus, Denmark). The analysis parameters used in this study were the same as those used in our previous study (Miyamoto et al., 2016a). To calculate the relative coverage increase (RCI), a pseudocount of 1 was inserted at each nucleotide position with zero coverage, and the coverage of the nth position was divided by the coverage of the (n-1)th position (*n*≥2). Positions with coverages lower than the median value of the coverage distribution for each RNA substrate were excluded from analysis. All five base sequences upstream and downstream of the base position with RCI>3 were selected as reference sequences. The ten sequences showing the highest RCI value were aligned using the WebLogo software. These sequence data have been submitted to the DDBJ database under the accession numbers DRA011779 (wild-type MazF-Da) and DRA011780 (MazF-Da mutants).

### Determination of the MazF-Da Cleavage Sequence *via* Fluorometric Assay

Fluorometric assays were performed as described in our previous study (Miyamoto et al., 2016a). Briefly, 0.5pmol of MazF-Da, its mutant, or RNase A was incubated with 20pmol of fluorescently labeled oligonucleotides (Supplementary Table S2) in 20μl of MazF reaction buffer. For the neutralization reaction, 0.5pmol of MazF-Da was preincubated with 5pmol of MazE-Da for 10min at 25°C, followed by the addition of 20pmol of fluorescently labeled oligonucleotides to the reaction buffer in a total volume of 20μl and incubation. All incubations were performed for 90min at 60°C, and fluorescence intensity was recorded every minute using a Light Cycler 480 system (Roche, Basel, Switzerland) with 483nm excitation and 533nm detection filters. All data were collected in triplicate and the average was calculated.

### Analysis of UACAAA Frequency in *Candidatus* Desulforudis audaxviator Coding Sequences

Statistical analysis was performed as described previously (Miyamoto et al., 2018). The protein-coding sequences of *D. audaxviator* were retrieved from the NCBI database. Coding sequence (CDS) data from 12 March 2020 were used for the analysis.

### Accession Numbers

The GenBank accession numbers were as follows: artificially designed RNAs: 500-2 (AB610940), 1000-1 (AB610944), 1000-2 (AB610945), 1000-3 (AB610946), 1000-4 (AB610947), 1000-5 (AB610948), 1500-1 (AB610949), and 2000-1 (AB610950); *Candidatus* Desulforudis audaxviator genome (NC_010424), MazE-Da (WP_012302901.1), and MazF-Da (WP_012302900.1).

## Results

### Identification of *Candidatus* Desulforudis audaxviator MazEF

**Figure 1:**
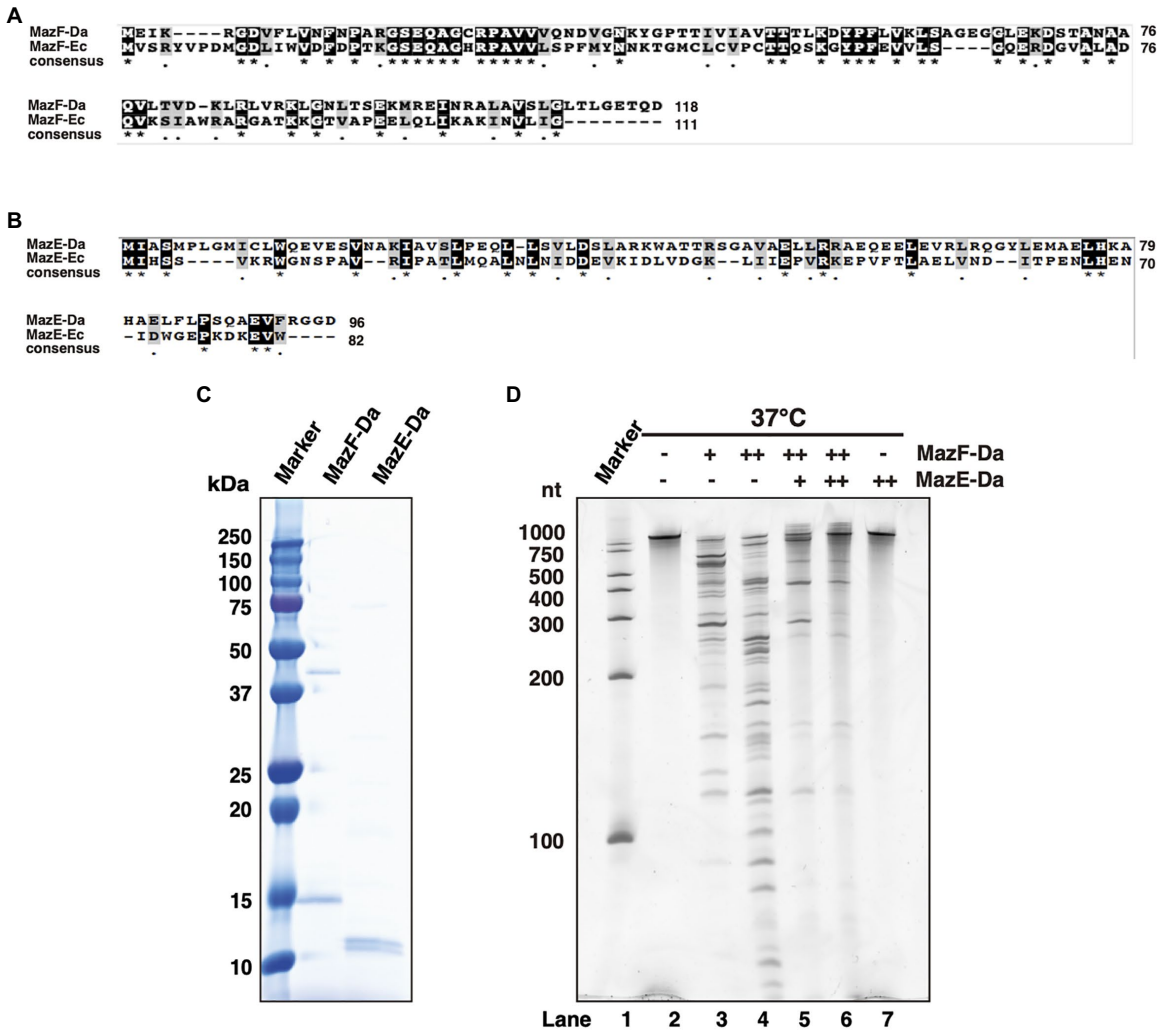
Characterization of MazEF-Da. Alignment of the **(A)** MazF-Da and MazF-Ec as well as the **(B)** MazE-Da and MazE-Ec amino acid sequences. For both **(A)** and **(B)**, identical amino acid residues are highlighted as black squares and highly similar amino acid residues are highlighted as gray squares. Asterisks indicate 100% identity between the two sequences and dots indicate 80% similarity between the two sequences. **(C)** The molecular weight and purity of MazE-Da and MazF-Da were assessed using SDS- PAGE and visualized using Coomassie Brilliant Blue (CBB) staining. **(D)** Substrate RNA (RNA 1500–1) was incubated at 37°C for 90min with MazE-Da and/or MazF-Da; lane 1, marker; lane 2, negative control without enzymes; lane 3, 1pmol of MazF-Da; lane 4, 10pmol of MazF-Da; lane 5, 10pmol of MazF-Da and 20pmol of MazE-Da; lane 6, 10pmol of MazF-Da and 40pmol of MazE-Da; lane 7, 40pmol of MazE-Da.

The MazF-Da toxin consisted of 118 amino acids, which exhibited 33.1% identity and 48% similarity to those of MazF-Ec (Figure 1A). The cognate antitoxin MazE-Daud1832 (MazE-Da) consisted of 96 amino acids, exhibiting 12.6% identity and 25.2% similarity to those of MazE-Ec (Figure 1B). Furthermore, MazEF-Da showed the following two characteristics of type II TA systems: (i) *mazE-Da* was present upstream of *mazF-Da*, and these genes constituted the operon structure; (ii) the two genes had an 11-bp overlapping sequence downstream of *mazE-Da* and upstream of *mazF-Da*.

To confirm whether MazEF-Da functions as a TA protein, the MazEF-Da proteins were expressed using an *E. coli* protein expression system. Although wild-type MazE-Da was successfully obtained (Figure 1C), wild-type MazF-Da was not. Due to unintended mutations in the open reading frame of the MazF-Da expression plasmid, only MazF-Da mutants were obtained. Thus, we utilized a cell-free protein synthesis system instead of *E. coli* (Shimizu et al., 2001, 2005; Shin and Noireaux, 2010) and successfully obtained mutation-free MazF-Da (Figure 1C). To investigate its ribonuclease activity, MazF-Da was mixed with a substrate RNA and incubated at 37°C. MazF-Da exhibited concentration-dependent cleavage activity against substrate RNA (Figure 1D). Next, to examine whether the enzymatic activity of MazF-Da was inhibited by MazE-Da, the two proteins were mixed at 25°C, followed by the addition of substrate RNA and incubation at 37°C. The enzymatic activity of MazF-Da was suppressed by MazE-Da in a dose-dependent manner (Figure 1D). These findings suggested that MazF-Da possessed ribonuclease activity that was neutralized by MazE-Da.

### Optimum Temperature for MazF-Da Enzyme Activity

**Figure 2:**
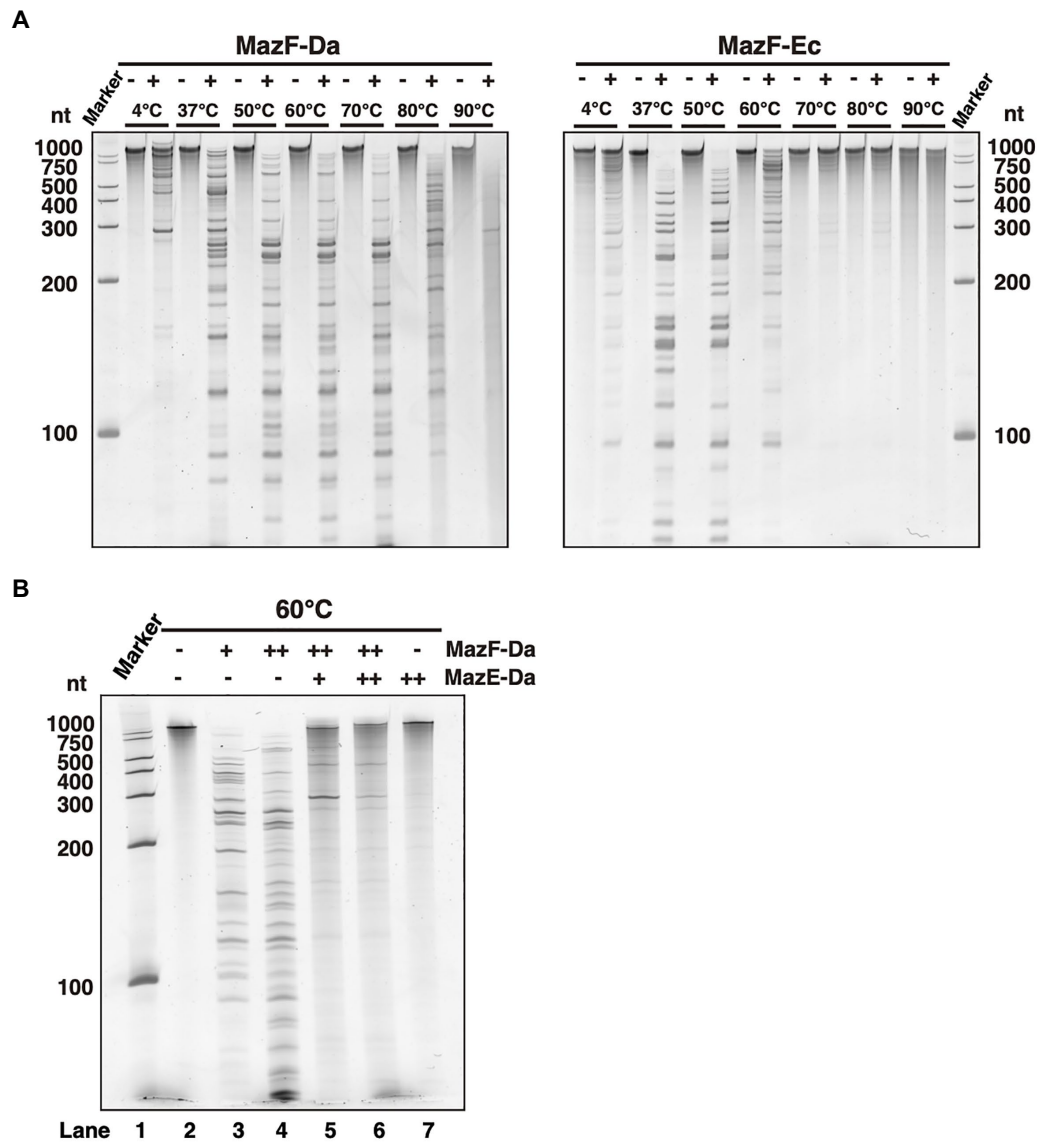
Enzyme activity of MazEF-Da at various temperatures. **(A)** MazF-Da (left side) and MazF-Ec (right side) were incubated with the substrate RNA at 4–90°C each. The resulting RNA fragments were separated on a 7.5% polyacrylamide gel containing 7M urea. **(B)** Substrate RNA was incubated at 60°C for 90min with MazE-Da and/or MazF-Da; lane 1, marker; lane 2, negative control without enzymes; lane 3, 1pmol of MazF-Da; lane 4, 10pmol of MazF-Da; lane 5, 10pmol of MazF-Da and 20pmol of MazE-Da; lane 6, 10pmol of MazF-Da and 40pmol of MazE-Da; lane 7, 40pmol of MazE-Da.

*Candidatus* Desulforudis audaxviator has been reported to live at environmental temperatures of approximately 60°C (Chivian et al., 2008; Karnachuk et al., 2019). To confirm whether the optimum temperature for MazF-Da activity is approximately the same as its environmental growth temperature, MazF-Da was incubated at temperatures ranging from 4 to 90°C for 10min, followed by the addition of substrate RNA and further incubation at each temperature for 60min. MazF-Da enzymatic activity was observed across 37–80°C temperature range, with the highest catalytic activity being seen at 50–70°C (Figure 2A). On the other hand, MazF-Ec activity was observed across the 37–60°C temperature range, with the highest catalytic activity being seen at approximately 37°C (Figure 2A). Both MazF-Da and MazF-Ec exhibited weak activity at 4°C (Figure 2A). Slight MazF-Da activity was also observed at 90°C; however, slight substrate degradation was also observed in the absence of the enzyme (Figure 2A).

Next, we assessed whether MazF-Da functions as a ribonuclease and MazE-Da as a suppressor at environmental growth temperature of 60°C. An increase in MazF-Da activity was observed, which was suppressed by MazE-Da in a dose-dependent manner (Figure 2B). Thus, we confirmed that MazF-Da had ribonuclease activity at 60°C and that MazE-Da could neutralize MazF-Da at that temperature.

### MazF-Da Preferentially Cleaved the UACAAA Sequence

**Figure 3:**
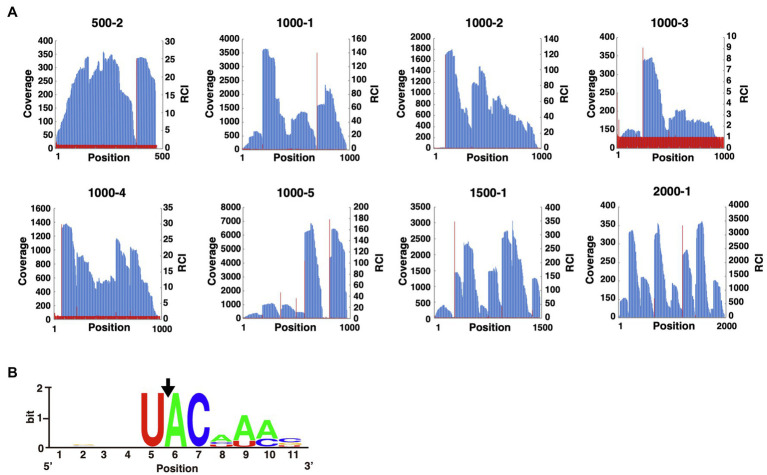
Analysis of the MazF-Da cleavage sequence. **(A)** The X-axis indicates the nth position from the 5'-terminus of the reference sequence. The left side of the Y-axis (blue bar) and the right side of the Y-axis (red line) indicate the coverage and the relative coverage increase (RCI) at each nth position, respectively. **(B)** WebLogo was used to visualize the aligned sequences upstream and downstream of the nucleotide positions with increased coverage. The position of the nucleotide with a significant increase in coverage was set to six on the X-axis. The sequences used for WebLogo alignment were the ten sequences with the highest RCI. The black arrow indicates the position of the MazF-Da cleavage site.

To identify MazF-Da recognition sequences, we conducted a massively parallel sequencing analysis (Miyamoto et al., 2016a). First, eight artificial synthetic RNAs (500-2, 1000-1, 1000-2, 1000-3, 1000-4, 1000-5, 1500-1, and 2000-1) were digested with MazF-Da at 60°C, and the cleavage sites were phosphorylated and barcoded. Next, we performed reverse transcription to produce cDNAs, attached adapters, and finally analyzed the cleavage sequence using next-generation sequencing. All read sequences were mapped to the reference RNA position. The coverage of the nth position and the RCI, that is, the ratio of the coverage at the nth position to that at the (n-1)th position, were then plotted on the Y-axis of the graphs (Figure 3A). From these graphs, we obtained the positions that fit the following two criteria: (i) the coverage was greater than or equal to the median value of coverage distribution for each RNA substrate (Supplementary Table S4); (ii) the RCI was greater than or equal to 3. The sequences, including five bases upstream and downstream of this extracted position, are listed. We selected the sequences with the 10 highest RCI values from the list (Supplementary Table S5) and visualized the similarity using WebLogo (Crooks et al., 2004). The sequence U^ACAAA (where ^ is the truncated site) emerged as a candidate cleavage sequence (Figure 3B).

To confirm whether MazF-Da cleaved the candidate recognition sequence, we prepared a fluorescently labeled DNA/RNA chimeric probe (Supplementary Table S2) and performed fluorescence assays (Wang and Hergenrother, 2007; Miyamoto et al., 2016a). This probe was a DNA/RNA chimeric oligonucleotide with five DNA adenine bases added to both ends of the RNA candidate cleavage sequence of MazF-Da. In addition, its 5' end was modified with 6-carboxyfluorescein (6-FAM) and the opposite 3' end with a black hole quencher (BHQ-1).

Normally, the fluorescence of 6-FAM at the 5' end of a fluorescent probe is quenched by BHQ-1 at the 3' end. However, when MazF causes cleavage of the target sequence within the fluorescent probe, 6-FAM is separated from BHQ-1 and thus it fluoresces. As the cleavage reaction of the probe proceeds, the amount of 6-FAM released from BHQ-1 increases, resulting in enhanced fluorescence intensity.

**Figure 4:**
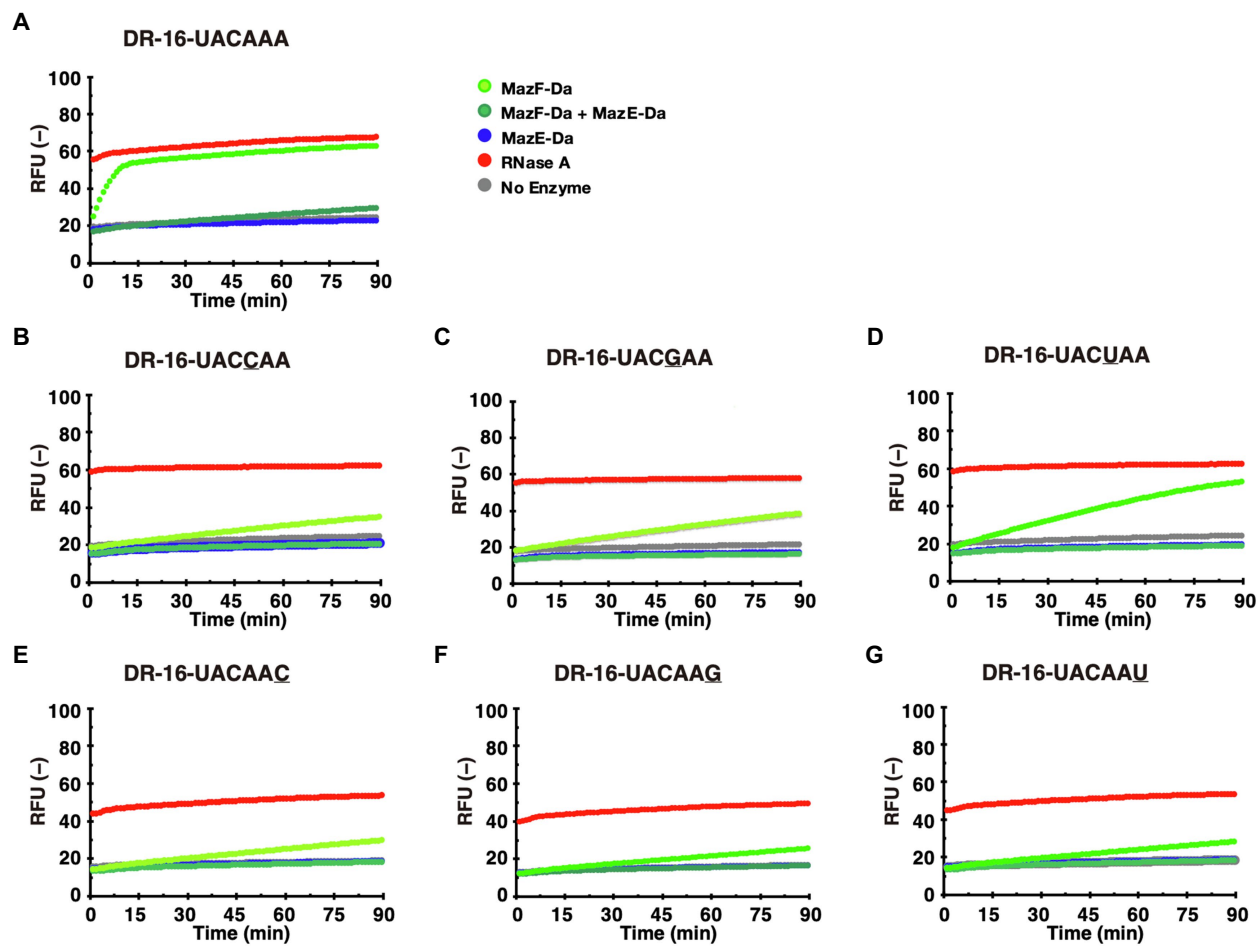
Identification of the MazF-Da cleavage sequence. MazF-Da (light-green), MazE-Da (blue), or MazF-Da preincubated with MazE-Da (green) were reacted with each of the following fluorescent probes (A–G): **(A)** DR-16-UACAAA, **(B)** DR-16-UACCAA, (C) DR-16-UACGAA, (D) DR-16-UACUAA, (E) DR-16-UACAAC, **(F)** DR-16-UACAAG, (G) DR-16-UACAAU. Fluorescence intensities in the presence of RNase A (red) and in the absence of enzymes (gray) were continuously assessed as control reactions.

The reaction of the candidate sequence UACAAA probe with MazF-Da at 60°C resulted in time-dependent cleavage of the probe by MazF-Da (Figure 4A). As shown in Figure 3B, the fourth adenine (A_4_) from the left of the UACAAA sequence was much less accentuated than the other bases in the UACAAA sequence. Thus, we determined whether this A_4_ was required for cleavage activity using the UACCAA, UACGAA, and UACUAA probes. MazF-Da showed weaker activity against UACUAA than UACAAA, and weaker activity against UACCAA and UACGAA than UACUAA (Figures 4B–D). Further, lower enzyme activity was observed with UACCAA than with the other three substrates (Figure 4B). Next, to determine whether MazF-Da is a six-base cutter, enzyme activity was assessed using the UACAAC, UACAAG, and UACAAU probes. The cleavage activity of MazF-Da with these probes was considerably weaker observed with UACAAA (Figures 4E–G), suggesting that A_6_ of UACAAA is important for MazF-Da enzyme activity. Next, to determine whether MazF-Da is a six- or a seven-base-specific sequence cutter, we assessed enzymatic activity using UACAAAA, UACAAAC, UACAAAAG, and UACAAAU probes with A_7_, C_7_, G_7_, or U_7_ added to UACAAA as the seventh base, respectively. MazF-Da exhibited comparable activity with UACAAA and with all the seven-nucleotide probes, suggesting that the seventh base was not included in the recognition sequence of MazF-Da (Supplementary Figure S1). These results indicated that MazF-Da was a six-base-recognizing ribonuclease that cleaved the UACAAA sequence most efficiently, and showed weak cleavage activity against UACCAA, UACGAA and UACUAA.

### Highly Conserved Amino Acid Moieties Were Crucial for Enzymatic Activity

**Figure 5:**
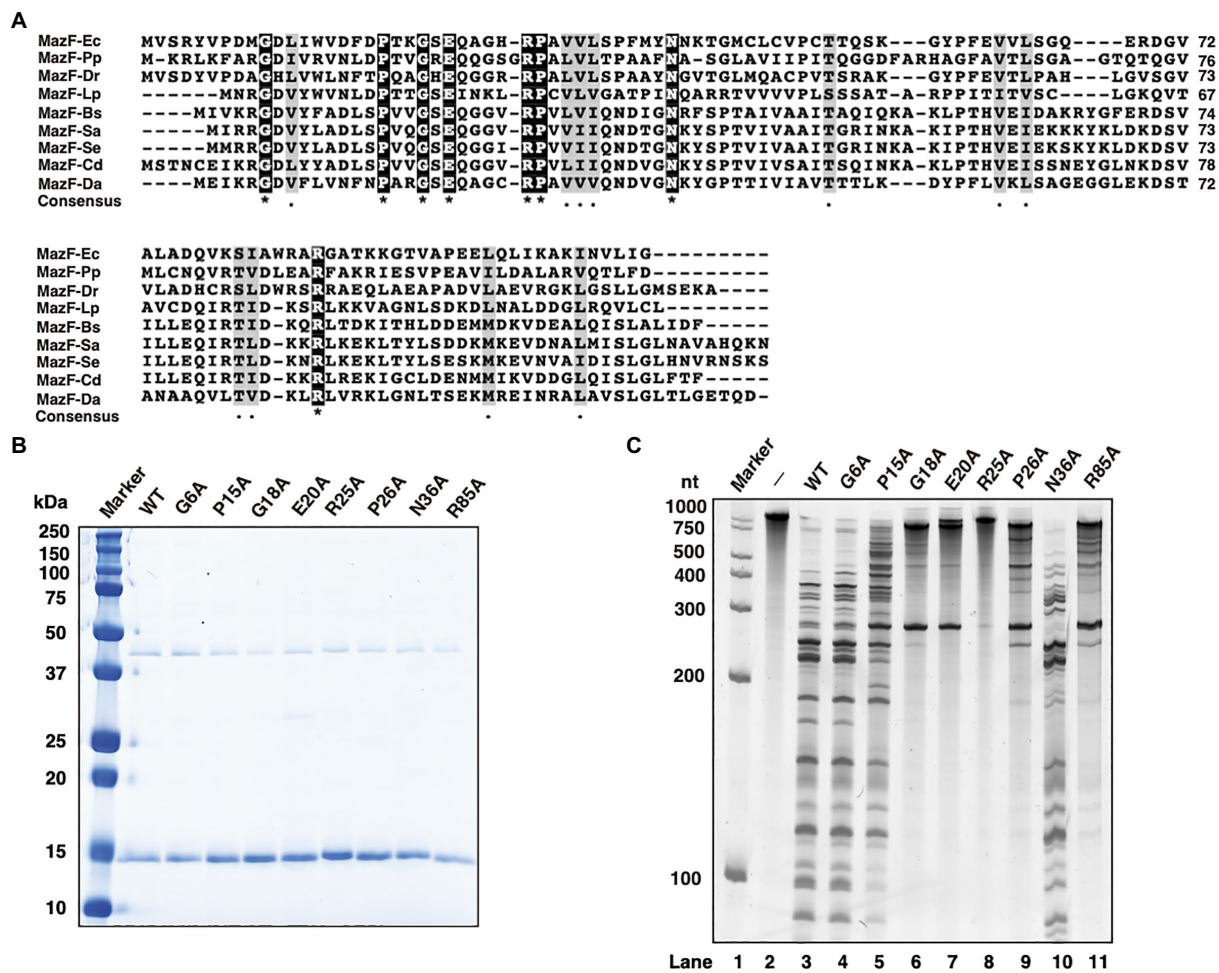
Characterization of MazF-Da mutants. **(A)** The amino acid sequences of MazF-Da and various MazFs were aligned as described below; Ec: *Escherichia coli*, Pp: *Pseudomonas putida*, Dr.: *Deinococcus radiodurans*, Lp: *Legionella pneumophila,* Bs: *Bacillus subtilis,* Sa: *Staphylococcus aureus*, Se: *Staphylococcus equorum*, Cd: *Clostridium difficile*, and Da: *Candidatus* Desulforudis audaxviator. Black squares indicate a perfect amino acid match and gray squares indicate a high degree of amino acid similarity. Asterisks indicate 100% identity between the nine sequences and dots indicate 80% similarity between the nine sequences. **(B)** The molecular weight and purity of the wild-type MazF-Da (WT) and of the MazF-Da mutants (G6A, P15A, G18A, E20A, R25A, P26A, N36A, and R85A) obtained using a cell-free protein synthesis system were analyzed using SDS-PAGE and visualized using CBB staining. **(C)** After the reaction of each MazF-Da mutant with the substrate RNA, the resultant digested RNA fragments were analyzed using urea gel. Lane 1, marker; lane 2, negative control without enzyme; lanes 3, wild type; lane 4, G6A; lane 5, P15A; lane 6, G18A; lane7, E20A; lane 8, R25A; lane 9, P26A; lane 10, N36A; lane 11, R85A.

The amino acid sequences of MazFs from several species, the cleavage sequences (ACA, UAC, AACU, UACA, and UACAU) of which are similar to the UACAAA recognition sequence of MazF-Da (Zhang et al., 2003b; Zhu et al., 2009; Park et al., 2011; Rothenbacher et al., 2012; Yamaguchi et al., 2012; Schuster et al., 2013; Miyamoto et al., 2016a,b, 2017, 2018; Shaku et al., 2018; Ishida et al., 2019; Aoi et al., 2020), were selected for multiple alignment. We identified Gly6 (G6), Pro15 (P15), Gly18 (G18), Glu20 (E20), Arg25 (R25), Pro26 (P26), Asn36 (N36), and Arg85 (R85) as highly conserved sites (Figure 5A).

To determine whether these amino acid sites were essential for MazF-Da ribonuclease activity, MazF-Da mutants with single mutations, such as G6A, P15A, G18A, E20A, R25A, P26A, N36A, and R85A, were prepared using a cell-free protein synthesis system. The yield of every MazF-Da point mutant was either comparable to or better than that of the wild type (Figure 5B), and the mutants’ cleavage activity against the substrate RNA was subsequently assessed (Figure 5C). The R25A mutant did not exhibit any catalytic activity against the substrate RNA, G18A and E20A showed little activity, P26A and R85A were weakly active, P15A was slightly less active than the wild type, G6A activity was similar to that of the wild type, and N36A exhibited slightly stronger activity than the wild type. These results suggested that G18, E20, and R25 were essential for the ribonuclease activity of MazF-Da.

### Amino Acid Residues Contributing to the Recognition of Cleavage Sites

**Figure 6:**
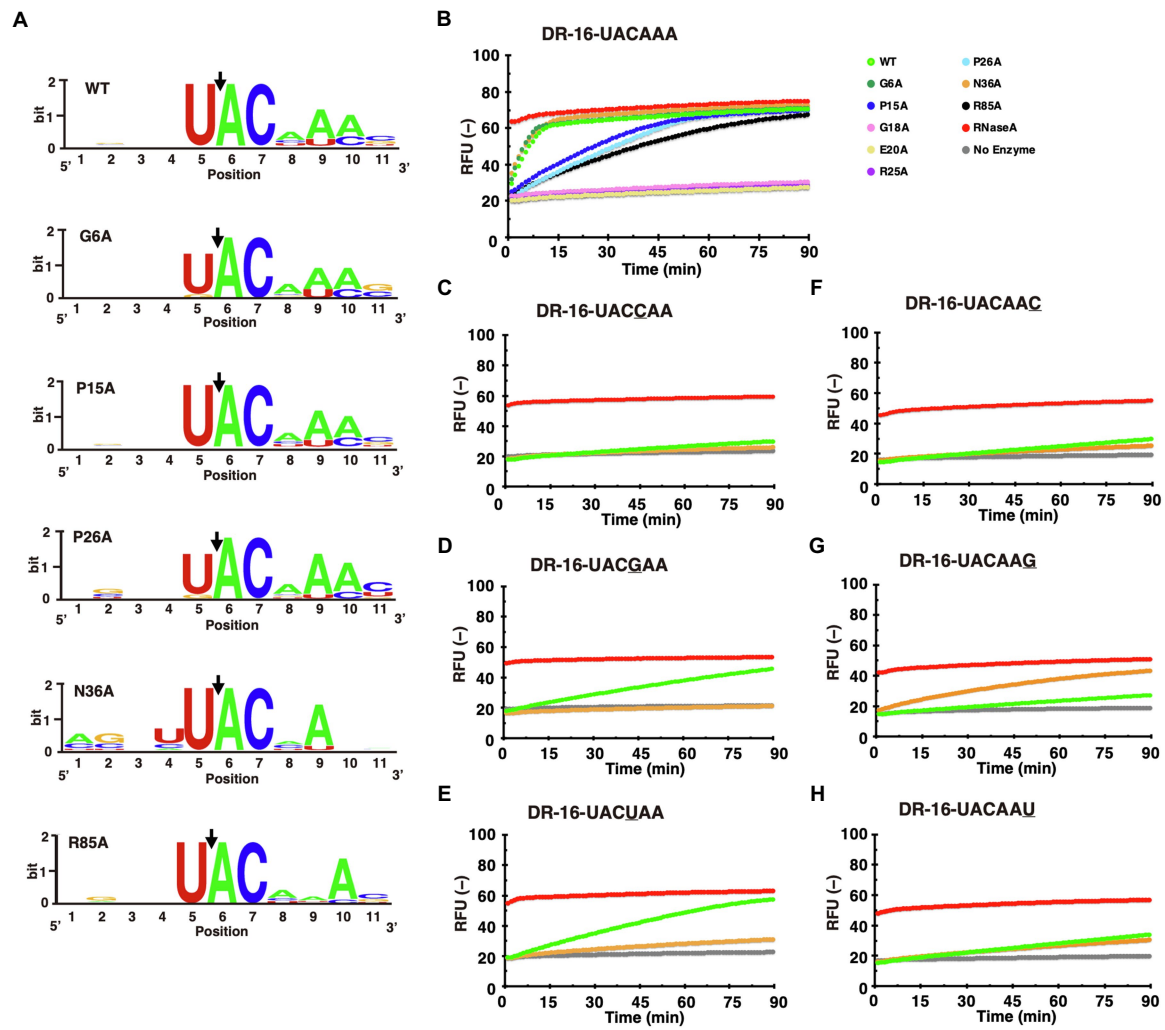
Comparison of recognition sequences between MazF-Da mutants. **(A)** For comparison of the recognition sequences between MazF-Da mutants (G6A, P15A, P26A, N36A, and R85A), the ten sequences with the highest RCI values were aligned in WebLogo. Black arrows indicate the location of truncation sites. **(B)** The UACAAA probe was reacted with each MazF-Da mutant (G6A, P15A, G18A, E20A, R25A, P26A, N36A, and R85A) and wild-type MazF-Da, and the fluorescence intensity was continuously measured. The activity of the wild-type and of each mutant at the time point of measurement is indicated as follows: WT, light-green; G6A, green; P15A, blue; G18A, pink; E20A, yellow; R25A, purple; P26A, sky blue; N36A, orange; R85A, black; RNase A, red; No enzyme, gray. Each fluorescent probe **(C**–**H)** was reacted with wild-type MazF-Da and the N36A mutant, and the fluorescence intensity was measured continuously. (C) DR-16-UACCAA, **(D)** DR-16-UACGAA, **(E)** DR-16-UACUAA, **(F)** DR-16-UACAAC, **(G)** DR-16-UACAAG, and (H) DR-16-UACAAU.

To investigate whether the recognition sequences of MazF-Da mutants were altered, we analyzed the cleavage sequence using next-generation sequencing. The G18A, E20A, and R25A mutants exhibited negligible enzymatic activity, as shown in Figure 5C. Hence, the recognition sequences could not be identified. UACAAA was present in the recognition sequences of the G6A, P15A, and P26A mutants, similar to that of the wild type (Figure 6A). The specificity of the N36A and R85A mutants for the recognition sequence was weaker than that of the wild type (Figure 6A).

First, we assessed the cleavage activity of mutants using the UACAAA probe, which is the recognition sequence of wild-type MazF-Da. The G6A and N36A mutants displayed the same activity as the wild type, whereas the P15A, P26A, and R85A mutants exhibited lower cleavage activity (Figure 6B). In contrast, G18A, E20A, and R25A did not cleave the probe (Figure 6B). Next, we examined whether the recognition sequences of the N36A and R85A mutants had changed. As shown in Figure 6A, in the N36A mutant, the A_4_ and A_6_ of UACAAA were not clearly visualized using WebLogo. Thus, we sought to identify the preferable nucleotide at the fourth and sixth positions of the target cleavage sequence in this mutant. We assessed the cleavage activity of potential recognition sequence candidates for N36A using the UACCAA, UACGAA, UACUAA, UACAAC, UACAAG, and UACAAU probes. Surprisingly, N36A cleaved the UACAAG sequence, against which the wild type or other mutants did not exhibit any enzymatic activity (Figures 6C–H). However, N36A did not show any activity with the UACGAA and UACUAA sequences, whereas the wild type did (Figures 6C–H). As shown in Figure 6A, in the R85A mutant, the A_5_ of UACAAA was not clearly visualized using in WebLogo, and we sought to identify the preferable nucleotide at the fifth position of the target sequence for cleavage by the mutant. We examined whether R85A cleaved the probes for recognition sequence candidates UACACA, UACAGA, and UACAUA. Negligible cleavage was observed, suggesting that the fifth adenine of UACAAA was essential for enzyme activity (Supplementary Figure S2). These results suggested that only the recognition sequence of the N36A mutant differed from that of the wild type.

### Amino Acid Moieties in MazF-Da Are Important for Its MazE-Da-Mediated Regulation

**Figure 7:**
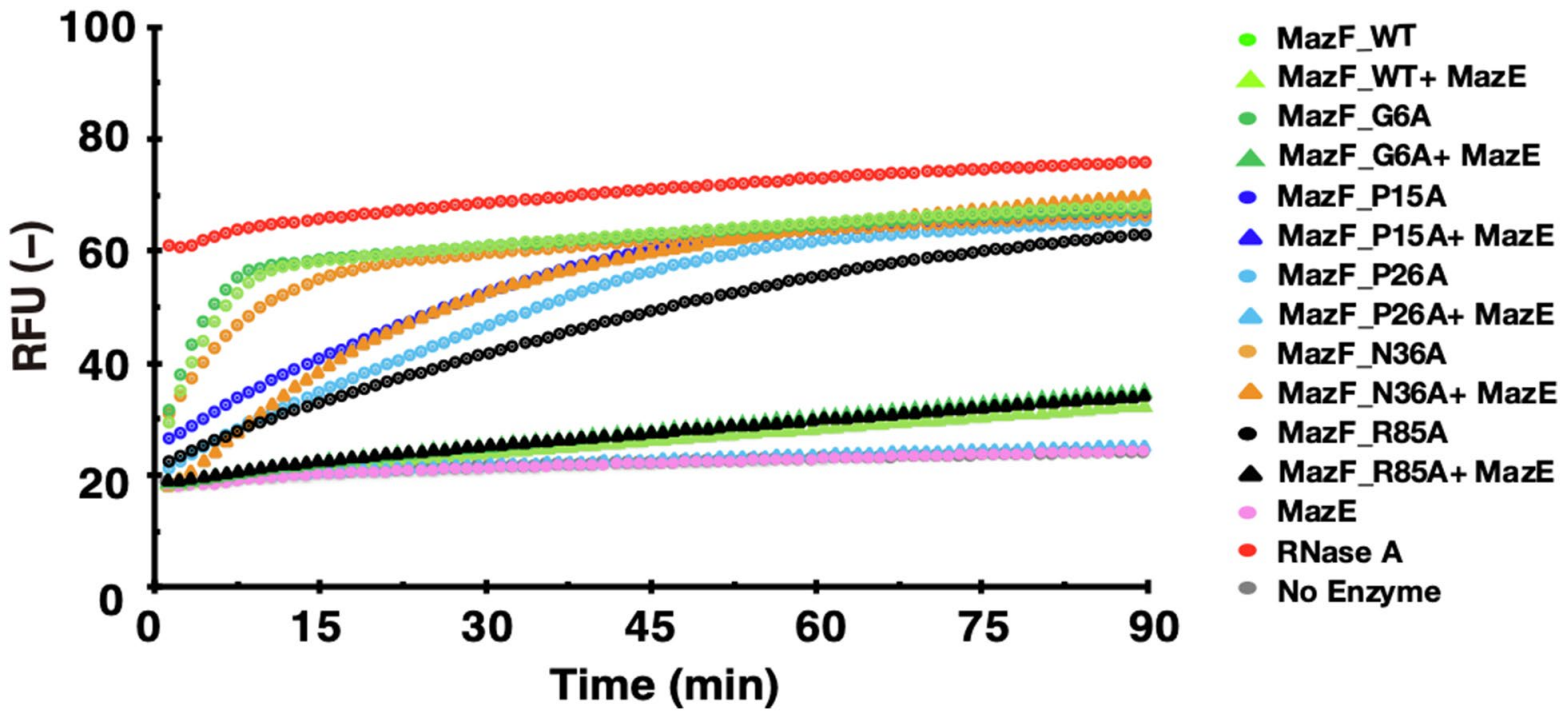
Neutralization of the activity of MazF-Da mutants by MazE-Da. The activity of MazF-Da or MazF-Da mutants against the UACAAA probe and the inhibition of activity by MazE-Da were measured. The fluorescence intensity was continuously measured. Circles indicate the MazF-Da or the MazF-Da mutant alone, and triangles indicate the MazF-Da or the Maz-Da mutant and the MazE-Da mixtures.

To investigate whether the highly conserved amino acids of MazF-Da were involved in the MazE-Da-mediated suppression of its activity, we performed neutralization assays using wild-type MazE-Da and MazF-Da mutants (G6A, P15A, P26A, N36A, and R85A) that exhibited cleavage activity (Figure 7). The enzyme activity of G6A was inhibited by MazE-Da to the same extent as that of the wild-type MazF-Da. The cleavage activities of P15A and P26A, which are slightly less active than the wild type, were almost completely inhibited by MazE-Da (Figure 7). Interestingly, though both the N36A and the wild-type MazF-Da exhibited almost the same enzymatic activities, MazE-mediated repression against N36A was weaker than that against the wild type (Figure 7). The cleavage activity of R85A was weaker than that of the wild type, whereas the inhibition of cleavage activity by MazE-Da was similar in both (Figure 7). These results suggested that the 36th and 85th amino acids of MazF-Da affected the binding of MazE-Da to MazF-Da and resulted in the loss of the MazE-Da inhibitory function.

## Discussion

In previous studies, crystallographic analyses of MazF isolated from different species have revealed the amino acid sites important for substrate RNA binding and catalytic activity (Kamada et al., 2003; Simanshu et al., 2013; Hoffer et al., 2017). However, the role of the well-conserved amino acids across various MazFs remains unclear. Our study revealed that MazF-Da is a unique six-base-recognizing cutter with cleavage activity at 60°C. Furthermore, we found that amino acids that are well-conserved between MazF-Da and other MazFs with similar recognition sequences are essential for its function.

### Expression Systems for MazEF Proteins

The unintended MazF-Da mutants, but not the wild-type protein, were expressed using the *E. coli* protein expression system. This may have resulted from the toxicity of MazF causing the host to prioritize the acquisition of less toxic MazF mutants. A method for MazF protein expression and purification using a host-independent protein expression system similar to that employed in this study has been previously reported (Zhang et al., 2003b). Another method is the use of the single protein production system (Suzuki et al., 2007), this system utilizes two types of plasmids; one is a plasmid expressing the MazF-Ec protein, which specifically cleaves the ACA sequence; the other is a plasmid in which the ACA sequence is removed from the open reading frame to express the target protein. By using both plasmids in conjunction to express MazF-Ec and the target protein simultaneously, the ACA sequence in the host-derived mRNAs is cleaved, thereby suppressing the expression of non-target proteins. As a result, only the target protein is efficiently expressed. Yet another method involves the co-expression of MazE and MazF in separate vectors to neutralize the toxicity of MazF to the host, thereby resulting in the efficient expression and acquisition of MazF (Sterckx et al., 2015; Dhanasingh et al., 2018).

In this study, we employed a cell-free protein synthesis system to obtain wild-type MazF-Da (Shimizu et al., 2001, 2005; Shin and Noireaux, 2010). The advantages of using this system for MazF expression are as follows: (i) it expresses proteins in a host-independent manner and is not affected by toxic effects on the host; (ii) by expressing and purifying proteins in a tube using the minimum amount of material required for protein expression, we can obtain highly pure proteins with almost no host-derived debris; (iii) many proteins can be easily expressed and purified simultaneously. However, not all proteins may be expressed in this manner. Therefore, providing a greater choice of expression methods for obtaining highly toxic proteins such as MazF is critical.

### Unique Enzymatic Properties of Various MazF Homologs

Our study revealed that MazF-Da has high endoribonuclease activity at 60°C, the same temperature as the natural habitat of *D. audaxviator*. In addition, MazF-Da exhibited enzymatic activity in the temperature range of 37–80°C, which was significantly wider than observed for MazF-Ec. This suggests that MazF-Da may maintain its enzymatic activity and function even under habitat temperature changes.

MazF obtained from the highly halophilic bacterium *Methanohalobium evestigatum* (MazFme) is not enzymatically active under high salinity conditions (Ishida et al., 2019). Moreover, MazEme did not inhibit the enzymatic activity of MazFme between 37 and 60°C (the temperature range of MazFme enzymatic activity) (Ishida et al., 2019). More recently, it was shown that MazF-Ec lost its cleavage activity against the ACA sequence when the adenine at the cleavage site of the recognition sequence was modified to N6-methyladenosine (m^6^A) (Imanishi et al., 2017). Furthermore, based on this property of MazF-Ec against the m^6^ACA sequence, a method for the quantitative analysis of the RNA m^6^A modification, which is important for eukaryotic development, physiology, and disease, has been reported previously (Garcia-Campos et al., 2019). These characteristics of MazF imply that its unique enzymatic properties may be conserved across various species. Elucidating the specific conditions required for MazF enzymatic activity, including optimal pH, temperature, salt concentration, and RNA modification, may lead to the development of novel research methods and applications.

### Relationship Between Catalytic Activity of MazF-Da and Its Highly Conserved Amino Acids

Crystal structure analysis of the complex of the uncleavable UUdUACAUAA substrate RNA and MazF-Bs revealed that Arg25 and Thr48 are involved in substrate RNA binding and that mutations in these amino acids result in loss of toxicity to *E. coli* (Simanshu et al., 2013). Moreover, Gly18 of MazF-Bs forms a hydrogen bond with the second adenine of UACAU through its Hoogsteen edge, whereas Pro26 forms a hydrogen bond with the first uracil of UACAU through its Watson-Crick edge, suggesting that they may play a critical role in substrate RNA binding (Simanshu et al., 2013). Furthermore, crystallographic analysis of the E24A mutant of MazF-Ec revealed that this mutation impairs substrate recognition, resulting in loss of cleavage activity (Li et al., 2006).

We identified G18, E20, and R25 of MazF-Da as the key amino acid residues involved in its enzymatic activity. These are consistent with residues previously reported to be involved in MazF substrate binding (Li et al., 2006; Simanshu et al., 2013). Although the P26 amino acid of MazF is also important for substrate binding (Simanshu et al., 2013), our results showed that the activity of the alanine-substituted mutant MazF-Da (P26A) did not decrease as much as that of the three above-mentioned amino acid mutants. However, the activity was almost lost upon replacement with aspartic acid (Supplementary Figures S3A-C). These results suggested that P26, as well as G18, E20, and R25, are essential for the enzymatic activity of MazF-Da, which is consistent with the residues previously reported to be critical for substrate binding in the crystal structures of MazF-Ec and MazF-Bs. As G18, E20, R25, and P26 of MazF-Da appear to be implicated in substrate binding, a decrease in affinity for the substrate may have resulted in the loss of cleavage activity. In contrast, although P15 and R85 have not been reported to be involved in substrate binding, the P15A and R85A mutants exhibited weaker cleavage activity than the wild type. These amino acids may be indirectly involved in substrate binding or may directly affect catalytic functions. The amino acid sites analyzed in our study were conserved across various MazF toxins with similar recognition sequences. Therefore, the examination of highly conserved amino acid sites in MazF toxins with similar recognition sequences may be beneficial for elucidating the amino acid sites important for MazF cleavage activity.

### Amino Acid Sites That Alter the Recognition Sequence

Ishida et al. (2013) reported that substitution of all arginine units in MazF-Bs with canavanine changed the recognition sequence from five-base UACAU to six-base UACAUA. Our massively parallel sequencing analysis data predicted that the point mutation at N36 changed the recognition sequence of wild-type MazF-Da from UACAAA to UACNAN. Investigation using a fluorescent probe with the predicted cleavage sequence revealed that the N36A mutant was active against the UACAAG sequence, but was completely inactive against UACGAA and UACUAA sequences, which were cleaved by the wild-type MazF-Da. In MazF toxins derived from other species, it has not been reported whether the amino acid corresponding to N36 of MazF-Da plays a critical role in substrate RNA binding or recognition sequence regulation. Our results suggest that the identification of key MazF amino acid residues related to the loosening and narrowing of sequence recognition may enable us to generate novel MazF proteins with new cleavable sequences.

### Predicting the Physiological Role of *D. audaxviator* MazF *in vivo*

A search revealed that there are 153 CDSs containing one or more MazF-Da main recognition sequence (UACAAA) motifs in *D. audaxviator* (Supplementary Table S6). These 153 CDSs include many kinds of genes, including the hydrogenase, transposase, and hypothetical proteins with unknown function. However, it is not known from *in vitro* studies whether the 153 CDSs, which are potential targets of MazF-Da, are in fact equally or preferentially regulated by *in vivo* MazF-Da. Recently, it has been reported that the growth rate and biomass formation of *D. audaxviator* can be increased by reducing the concentration of calcium and phosphate in the medium and adding spermidine (Lukina and Karnachuk, 2021). Because the culture conditions of *D. audaxviator* have been established, it may be possible to verify our findings in this study by *in vivo* experiments using cultured *D. audaxviator* in the future.

In conclusion, the function of MazF isolated from the extremophilic microorganism *D. audaxviator* was clarified through *in vitro* analysis. We also generated MazF mutants with alterations in several highly conserved amino acids and identified residues that are important for the function of MazF-Da. Although *in vitro* functional analysis presents limitations in understanding how MazF regulates the translation of its target genes *in vivo*, the identification of MazF-Da recognition sequences *in vitro* will allow us to infer its function *in vivo* and to partially understand the ecological role of *D. audaxviator* within its habitat.

## Data Availability Statement

The datasets presented in this study can be found in online repositories. The names of the repository/repositories and accession number(s) can be found at: https://www.ddbj.nig.ac.jp/, DRA011779 and DRA011780.

## Author Contributions

HT-I, NN, and AY conceived and designed the experiments and reviewed and edited the manuscript. HT-I and MT performed the experiments. HT-I, MT, and AY analyzed the data. HT-I wrote the original draft of the manuscript. All authors contributed to the article and approved the submitted version.

## Funding

This work was supported by the Japan Society for the Promotion of Science KAKENHI Grant Nos. 19K06555 and 19H02879.

## Conflict of Interest

The authors declare that the research was conducted in the absence of any commercial or financial relationships that could be construed as a potential conflict of interest.

## Publisher’s Note

All claims expressed in this article are solely those of the authors and do not necessarily represent those of their affiliated organizations, or those of the publisher, the editors and the reviewers. Any product that may be evaluated in this article, or claim that may be made by its manufacturer, is not guaranteed or endorsed by the publisher.
