# Supplementary material for: Conserved Amino Acid Moieties of Candidatus Desulforudis audaxviator MazF Determine Ribonuclease Activity and Specificity
Source: Front Microbiol. 2021 Nov 11;12:748619. doi: 10.3389/fmicb.2021.748619 (PMC8634880; doi:10.3389/fmicb.2021.748619)
Supplement: Supplementary file 1 [file Data_Sheet_1.DOCX]

Supplementary Material

**Conserved amino acid moieties of *Candidatus* Desulforudis audaxviator MazF determine ribonuclease activity and specificity**

Hiroko Tamiya-Ishitsuka, Masako Tsuruga, Naohiro Noda^*^, and Akiko Yokota^*^

*** Correspondence:**Naohiro Noda
E-mail: [noda-naohiro@aist.go.jp](mailto:noda-naohiro@aist.go.jp)

Akiko Yokota
E-mail: [akiko-yokota@aist.go.jp](mailto:akiko-yokota@aist.go.jp)

**Supplementary Table S1:** Codon-optimized *mazE-Da* and *mazF-Da* genes sequence for *E. coli*

| Optimized *mazE-Da* sequence | ATGATCGCGAGCATGCCGCTGGGTATGATTTGCCTGTGGCAGGAAGTGGAAAGCGTTAACGCGAAGATCGCTGTGAGCCTGCCGGAGCAACTGCTGAGCGTTCTGGACAGCCTGGCGCGTAAATGGGCGACCACCCGTAGCGGTGCGGTGGCGGAACTGCTGCGTCGTGCGGAGCAGGAAGAGCTGGAAGTTCGTCTGCGTCAAGGCTACCTGGAGATGGCGGAACTGCACAAAGCGCATGCGGAGCTGTTCCTGCCGAGCCAGGCGGAAGTGTTTCGTGGTGGCGATCTCGAGCACCACCACCACCACCACTGA |
| --- | --- |
| Optimized *mazF-Da* sequence | ATGGAAATCAAACGCGGTGATGTCTTTCTGGTGAACTTTAATCCGGCCCGTGGCAGCGAACAGGCAGGTTGTCGTCCGGCGGTGGTGGTTCAGAACGATGTGGGCAATAAATATGGTCCGACCACCATTGTGATCGCGGTTACCACCACCCTGAAAGATTATCCGTTTCTGGTTAAACTGAGCGCGGGCGAAGGCGGTCTGGAAAAAGATAGCACCGCAAATGCCGCACAGGTGCTGACCGTTGATAAACTGCGTCTGGTGCGCAAACTGGGTAATCTGACCAGCGAAAAAATGCGTGAAATCAACCGTGCTCTGGCAGTGTCCCTGGGTCTGACGCTGGGCGAAACGCAAGATCTCGAGCACCACCACCACCACCACTGA |

**Supplementary Table S2:** Fluorogenic oligonucleotides used in this study.

| **Fluorescence probe** | **Probe sequence (5′ – 3′) ^a^** |
| --- | --- |
| DR-16-UACAAA | AAAAAUACAAAAAAAA |
| DR-16-UACCAA | AAAAAUACCAAAAAAA |
| DR-16-UACGAA | AAAAAUACGAAAAAAA |
| DR-16-UACUAA | AAAAAUACUAAAAAAA |
| DR-16-UACAAC | AAAAAUACAACAAAAA |
| DR-16-UACAAG | AAAAAUACAAGAAAAA |
| DR-16-UACAAU | AAAAAUACAAUAAAAA |
| DR-17-UACAAAA | AAAAAUACAAAAAAAAA |
| DR-17-UACAAAC | AAAAAUACAAACAAAAA |
| DR-17-UACAAAG | AAAAAUACAAAGAAAAA |
| DR-17-UACAAAU | AAAAAUACAAAUAAAAA |
| DR-16-Um^6^ACAAA | AAAAAUm^6^ACAAAAAAAA |

^a^ Underlined letters represent RNA nucleotides, whereas other letters represent DNA nucleotides.

**Supplementary Table S3:** Primers used to create MazF-Da mutants in this study.

| **Primer name** | **Primer sequence (5′ – 3′)** |
| --- | --- |
| MazF-Da G6A Fw | AAACGCGCTGATGTCTTTCTGGTGAAC |
| MazF-Da G6A Rv | GACATCAGCGCGTTTGATTTCCATTGA |
| MazF-Da G6D Fw | AAACGCGATGATGTCTTTCTGGTGAAC |
| MazF-Da G6D Rv | GACATCATCGCGTTTGATTTCCATTGA |
| MazF-Da P15A Fw | TTTAATGCGGCCCGTGGCAGCGAACAG |
| MazF-Da P15A Rv | ACGGGCCGCATTAAAGTTCACCAGAAA |
| MazF-Da P15D Fw | TTTAATGATGCCCGTGGCAGCGAACAG |
| MazF-Da P15D Rv | ACGGGCATCATTAAAGTTCACCAGAAA |
| MazF-Da G18A Fw | GCCCGTGCGAGCGAACAGGCAGGTTGT |
| MazF-Da G18A Rv | TTCGCTCGCACGGGCCGGATTAAAGTT |
| MazF-Da E20A Fw | GGCAGCGCACAGGCAGGTTGTCGTCCG |
| MazF-Da E20A Rv | TGCCTGTGCGCTGCCACGGGCCGGATT |
| MazF-Da R25A Fw | GGTTGTGCACCGGCGGTGGTGGTTCAG |
| MazF-Da R25A Rv | CGCCGGTGCACAACCTGCCTGTTCGCT |
| MazF-Da P26A Fw | TGTCGTGCGGCGGTGGTGGTTCAGAAC |
| MazF-Da P26A Rv | CACCGCCGCACGACAACCTGCCTGTTC |
| MazF-Da P26D Fw | TGTCGTGATGCGGTGGTGGTTCAGAAC |
| MazF-Da P26D Rv | CACCGCATCACGACAACCTGCCTGTTC |
| MazF-Da N36A Fw | GTGGGCGCTAAATATGGTCCGACCACC |
| MazF-Da N36A Rv | ATATTTAGCGCCCACATCGTTCTGAAC |
| MazF-Da R85A Fw | AAACTGGCACTGGTGCGCAAACTGGGT |
| MazF-Da R85A Rv | CACCAGTGCCAGTTTATCAACGGTCAG |

The primers were designed to introduce mutations in the pET-24a(+)-*mazF-Da* plasmid (the mutated regions are underlined).

**Supplementary Table S4:** Median values of coverage distribution for each RNA substrate

| RNA | 500-2 | 1000-1 | 1000-2 | 1000-3 | 1000-4 | 1000-5 | 1500-1 | 2000-1 |
| --- | --- | --- | --- | --- | --- | --- | --- | --- |
| Median | 272 | 1133 | 702 | 285 | 633 | 874 | 1135 | 1792 |

**Supplementary Table S5:** Top 10 sequences with the highest RCI values.

| Rank | RNA | Position | RCI | Coverage | Sequence ^a^ |
| --- | --- | --- | --- | --- | --- |
| 1 | 2000-1 | 1210 | 3341 | 3341 | AGGCUACAAAG |
| 2 | 2000-1 | 683 | 706 | 4238 | AGUUUACAUAC |
| 3 | 1500-1 | 286 | 347 | 1391 | GGAAUACAAAU |
| 4 | 1000-5 | 831 | 178 | 4292 | GUGGUACAAAU |
| 5 | 1000-1 | 715 | 140 | 1542 | UGCUUACCAAC |
| 6 | 1000-2 | 109 | 118 | 1663 | ACCAUACAACC |
| 7 | 1000-5 | 592 | 104 | 6042 | CCCAUACAACC |
| 8 | 1500-1 | 961 | 41 | 2452 | GACUUACCUAG |
| 9 | 1000-4 | 69 | 29 | 1288 | CCAUUACUACG |
| 10 | 1500-1 | 1397 | 27 | 1223 | CGUCUACAAGC |

^a^ Underlined bases represent locations with a significant increase in coverage.

**Supplementary Table S6:** Protein-coding sequences containing UACAAA more than twice.

| **locus** | **location** | **gene product** | **UACAAA** |
| --- | --- | --- | --- |
| DAUD_RS09525 | 1983090..1985297 | hydrogenase_iron-sulfur_subunit | 3 |
| DAUD_RS00565 | 110679..112979 | CO dehydrogenase/acetyl-CoA_synthase_complex_subunit_epsilon | 2 |
| DAUD_RS00850 | 181126..182457 | transposase | 2 |
| DAUD_RS00935 | 198025..199356 | transposase | 2 |
| DAUD_RS01150 | 242044..243375 | transposase | 2 |
| DAUD_RS01165 | 251185..252516 | transposase | 2 |
| DAUD_RS01355 | 273907..275238 | transposase | 2 |
| DAUD_RS02080 | 441776..443107 | transposase | 2 |
| DAUD_RS02210 | 467206..468537 | transposase | 2 |
| DAUD_RS02230 | 471954..473285 | transposase | 2 |
| DAUD_RS02975 | 622204..623535 | transposase | 2 |
| DAUD_RS03990 | 825126..826850 | IS1182_family_transposase | 2 |
| DAUD_RS04230 | 886156..886728 | hypothetical_protein | 2 |
| DAUD_RS06295 | 1305752..1307083 | transposase | 2 |
| DAUD_RS07565 | 1575230..1577440 | CDC48_family_AAA_ATPase | 2 |
| DAUD_RS07625 | 1584594..1585925 | transposase | 2 |
| DAUD_RS07630 | 1586391..1587722 | transposase | 2 |
| DAUD_RS08775 | 1831062..1832393 | transposase | 2 |
| DAUD_RS08860 | 1846401..1847732 | transposase | 2 |
| DAUD_RS09155 | 1914303..1915298 | type_I-B_CRISPR-associated_endonuclease_Cas1 | 2 |
| DAUD_RS09215 | 1927051..1928874 | type III-B CRISPR-associated protein Cas10/Cmr2 | 2 |
| DAUD_RS11125 | 2307205..2308536 | transposase | 2 |
| DAUD_RS00120 | 23472..24464 | hypothetical_protein | 1 |
| DAUD_RS00140 | 27382..28917 | hypothetical_protein | 1 |
| DAUD_RS00145 | 28947..30455 | hypothetical_protein | 1 |
| DAUD_RS00155 | 31450..32919 | hypothetical_protein | 1 |
| DAUD_RS00165 | 33609..34820 | DegT/DnrJ/EryC1/StDAUD_RS_aminotransferase_family_protein | 1 |
| DAUD_RS00170 | 35052..36725 | hypothetical_protein | 1 |
| DAUD_RS00390 | 72773..73408 | 50S_ribosomal_protein_L25 | 1 |
| DAUD_RS00560 | 108495..110669 | formate_dehydrogenase_subunit_alpha | 1 |
| DAUD_RS11785 | 114410..115543 | NADH-quinone_oxidoreductase_subunit_NuoE | 1 |
| DAUD_RS00590 | 117535..120531 | molybdopterin-dependent_oxidoreductase | 1 |
| DAUD_RS00595 | 120667..121866 | CO dehydrogenase/CO-methylating_acetyl-CoA_synthase_complex_subunit_beta | 1 |
| DAUD_RS00600 | 121918..123276 | acetyl-CoA decarbonylase/synthase_complex_subunit_gamma | 1 |
| DAUD_RS00645 | 131154..132065 | quinolinate_synthase_NadA | 1 |
| DAUD_RS00675 | 137088..138092 | tRNA_dihydrouridine_synthase_DusB | 1 |
| DAUD_RS00890 | 189949..191061 | [FeFe]_hydrogenase_H-cluster_radical_SAM_maturase_HydE | 1 |
| DAUD_RS01105 | 236033..236497 | type_II_toxin-antitoxin_system_VapC_family_toxin | 1 |
| DAUD_RS01155 | 243762..247313 | DNA-directed_RNA_polymerase_subunit_beta | 1 |
| DAUD_RS01160 | 247379..250843 | DNA-directed_RNA_polymerase_subunit_beta' | 1 |
| DAUD_RS01250 | 262377..262745 | 50S_ribosomal_protein_L14 | 1 |
| DAUD_RS01275 | 264572..265117 | 50S_ribosomal_protein_L6 | 1 |
| DAUD_RS01385 | 282484..283995 | S-layer_protein | 1 |
| DAUD_RS01510 | 309036..310151 | S41_family_peptidase | 1 |
| DAUD_RS01580 | 326904..327788 | RNase_adapter_RapZ | 1 |
| DAUD_RS01620 | 335442..337490 | sodium-translocating_pyrophosphatase | 1 |
| DAUD_RS01640 | 340457..341650 | TRC40/GET3/ADAUD_RSA_family_transport-energizing_ATPase | 1 |
| DAUD_RS01945 | 403178..404122 | diguanylate_cyclase | 1 |
| DAUD_RS01950 | 404199..404522 | MoaD/ThiS_family_protein | 1 |
| DAUD_RS01970 | 409530..413780 | TIGR02680_family_protein | 1 |
| DAUD_RS02055 | 434695..437457 | DEAD/DEAH_box_helicase | 1 |
| DAUD_RS02065 | 437138..439942 | DUF1998_domain-containing_protein | 1 |
| DAUD_RS11420 | 457674..460235 | diguanylate_cyclase | 1 |
| DAUD_RS02180 | 463823..464836 | hypothetical_protein | 1 |
| DAUD_RS11425 | 478131..478733 | hypothetical_protein | 1 |
| DAUD_RS02585 | 541144..544587 | DUF3883_domain-containing_protein | 1 |
| DAUD_RS02640 | 559443..559658 | type_II_toxin-antitoxin_system_HicB_family_antitoxin | 1 |
| DAUD_RS02690 | 567958..569583 | PAS_domain-containing_protein | 1 |
| DAUD_RS11915 | 575328..577910 | diguanylate_cyclase | 1 |
| DAUD_RS02810 | 592996..594693 | DNA_mismatch_repair_endonuclease_MutL | 1 |
| DAUD_RS02825 | 596474..596725 | RNA_chaperone_Hfq | 1 |
| DAUD_RS03030 | 634666..635610 | DNA_polymerase_domain-containing_protein | 1 |
| DAUD_RS03180 | 666169..667179 | 2-hydroxyglutaryl-CoA_dehydratase | 1 |
| DAUD_RS03305 | 688736..690139 | aspartate_aminotransferase_family_protein | 1 |
| DAUD_RS03475 | 723178..724581 | circadian_clock_protein_KaiC | 1 |
| DAUD_RS03505 | 728868..730262 | ISNCY-like_element_ISCde2_family_transposase | 1 |
| DAUD_RS11480 | 739597..740511 | copper_amine_oxidase_N-terminal_domain-containing_protein | 1 |
| DAUD_RS03565 | 741574..743211 | DNA_methylase | 1 |
| DAUD_RS03570 | 743624..744772 | hypothetical_protein | 1 |
| DAUD_RS03595 | 749188..750333 | transposase | 1 |
| DAUD_RS03805 | 785354..786898 | hypothetical_protein | 1 |
| DAUD_RS03835 | 791120..791413 | hypothetical_protein | 1 |
| DAUD_RS03870 | 795374..795616 | type_II_toxin-antitoxin_system_HicB_family_antitoxin | 1 |
| DAUD_RS11995 | 798042..800684 | diguanylate_cyclase | 1 |
| DAUD_RS12005 | 816760..816963 | transposase | 1 |
| DAUD_RS03975 | 820357..822096 | adenylyl-sulfate_reductase_subunit_alpha | 1 |
| DAUD_RS03995 | 827685..828668 | nuclease | 1 |
| DAUD_RS04010 | 833593..835161 | DUF262_domain-containing_protein | 1 |
| DAUD_RS04085 | 854641..855060 | hypothetical_protein | 1 |
| DAUD_RS04235 | 886712..888187 | AAA_family_ATPase | 1 |
| DAUD_RS04255 | 891631..892776 | transposase | 1 |
| DAUD_RS04285 | 898759..902085 | maltose_alpha-D-glucosyltransferase | 1 |
| DAUD_RS04290 | 902413..903141 | hypothetical_protein | 1 |
| DAUD_RS04345 | 914060..915127 | glycosyltransferase_family_4_protein | 1 |
| DAUD_RS04840 | 1019345..1020973 | prepilin-type N-terminal cleavage/methylation_domain-containing_protein | 1 |
| DAUD_RS05005 | 1049691..1050062 | type_II_secretion_system_protein | 1 |
| DAUD_RS05230 | 1090888..1093344 | NTP_transferase_domain-containing_protein | 1 |
| DAUD_RS05280 | 1104452..1107679 | DNA_polymerase_III_subunit_alpha | 1 |
| DAUD_RS05365 | 1121038..1121307 | hypothetical_protein | 1 |
| DAUD_RS05495 | 1147655..1148503 | menaquinone_biosynthesis_protein | 1 |
| DAUD_RS05560 | 1160959..1161339 | 4Fe-4S_dicluster_domain-containing_protein | 1 |
| DAUD_RS05565 | 1161481..1162074 | formylmethanofuran_dehydrogenase_subunit_E | 1 |
| DAUD_RS05810 | 1207844..1208476 | DUF502_domain-containing_protein | 1 |
| DAUD_RS05845 | 1213631..1214524 | TIGR00269_family_protein | 1 |
| DAUD_RS06105 | 1266219..1269851 | cobaltochelatase_subunit_CobN | 1 |
| DAUD_RS06135 | 1275724..1277160 | ABC_transporter_substrate-binding_protein | 1 |
| DAUD_RS06290 | 1304812..1305195 | recombinase_family_protein | 1 |
| DAUD_RS06370 | 1319697..1320938 | AAA_family_ATPase | 1 |
| DAUD_RS04840 | 1330156..1331121 | SpoIID/LytB_domain-containing_protein | 1 |
| DAUD_RS06430 | 1336632..1337192 | RNA_polymerase_sigma_factor | 1 |
| DAUD_RS06545 | 1370179..1371174 | CRISPR_system_precrRNA_processing_endoribonuclease_RAMP_protein_Cas6 | 1 |
| DAUD_RS06620 | 1387799..1388257 | DUF441_domain-containing_protein | 1 |
| DAUD_RS06680 | 1398446..1399948 | glycogen_synthase_GlgA | 1 |
| DAUD_RS06845 | 1430626..1431858 | protein_translocase_subunit_SecD | 1 |
| DAUD_RS06925 | 1444916..1445587 | sporulation_protein_YunB | 1 |
| DAUD_RS07010 | 1465805..1467712 | threonine--tRNA_ligase | 1 |
| DAUD_RS07015 | 1468067..1468693 | DUF445_family_protein | 1 |
| DAUD_RS07345 | 1534979..1535290 | 50S_ribosomal_protein_L21 | 1 |
| DAUD_RS07410 | 1547347..1548171 | rod_shape-determining_protein_MreC | 1 |
| DAUD_RS07740 | 1607917..1608096 | hypothetical_protein | 1 |
| DAUD_RS07945 | 1647101..1647775 | HEAT_repeat_domain-containing_protein | 1 |
| DAUD_RS08135 | 1689274..1689549 | hypothetical_protein | 1 |
| DAUD_RS04840 | 1710085..1710819 | bifunctional phosphoribosyl-AMP cyclohydrolase/phosphoribosyl-ATP_diphosphatase_HisIE | 1 |
| DAUD_RS08290 | 1721105..1722514 | amidophosphoribosyltransferase | 1 |
| DAUD_RS08295 | 1722522..1723256 | phosphoribosylaminoimidazolesuccinocarboxamide_synthase | 1 |
| DAUD_RS08390 | 1742795..1743235 | pmethyl-viologen-reducing_hydrogenase_subunit_delta | 1 |
| DAUD_RS08480 | 1758982..1761822 | AAA_family_ATPase | 1 |
| DAUD_RS08490 | 1766919..1772708 | hypothetical_protein | 1 |
| DAUD_RS08500 | 1773659..1773997 | hypothetical_protein | 1 |
| DAUD_RS11670 | 1819934..1823458 | GAF_domain-containing_protein | 1 |
| DAUD_RS08785 | 1833596..1834594 | flagellar_motor_switch_protein_FliM | 1 |
| DAUD_RS08950 | 1861443..1862606 | UDP-4-amino-4_6-dideoxy-N-acetyl-beta-L-altrosamine_transaminase | 1 |
| DAUD_RS08990 | 1869148..1870200 | UDP-N-acetylglucosamine_4_6-dehydratase_(inverting) | 1 |
| DAUD_RS09005 | 1872987..1874246 | flagellin | 1 |
| DAUD_RS09085 | 1888323..1888637 | hypothetical_protein | 1 |
| DAUD_RS09135 | 1900969..1902363 | ISNCY-like_element_ISCde2_family_transposase | 1 |
| DAUD_RS09140 | 1904574..1905968 | ISNCY-like_element_ISCde2_family_transposase | 1 |
| DAUD_RS09145 | 1912286..1913680 | ISNCY-like_element_ISCde2_family_transposase | 1 |
| DAUD_RS09230 | 1930410.._1930583 | restriction_endonuclease | 1 |
| DAUD_RS09340 | 1944598..1945557 | cobalamin_biosynthesis_protein_CobD | 1 |
| DAUD_RS09405 | 1957163..1957453 | energy-coupling_factor_ABC_transporter_substrate-binding_protein | 1 |
| DAUD_RS09545 | 1989880..1990332 | YlbF_family_regulator | 1 |
| DAUD_RS09665 | 2014089..2015612 | hypothetical_protein | 1 |
| DAUD_RS09670 | 2015590..2016240 | IS607_family_transposase | 1 |
| DAUD_RS09715 | 2021205..2022782 | DNA_recombination_protein_RmuC | 1 |
| DAUD_RS09730 | 2024845..2026545 | NADH_dehydrogenase | 1 |
| DAUD_RS09740 | 2027909..2030581 | pyruvate__phosphate_dikinase | 1 |
| DAUD_RS09745 | 2030743..2031585 | fumarate_hydratase | 1 |
| DAUD_RS09760 | 2032922..2034709 | fumarate_reductase_flavoprotein_subunit | 1 |
| DAUD_RS09895 | 2062281..2063132 | phosphate_ABC_transporter_substrate-binding_protein | 1 |
| DAUD_RS09900 | 2063327..2064049 | metallophosphoesterase_family_protein | 1 |
| DAUD_RS10010 | 2079502..2079696 | hypothetical_protein | 1 |
| DAUD_RS10250 | 2125478..2126641 | amidohydrolase | 1 |
| DAUD_RS10335 | 2145837..2146985 | anti-sigma_factor_domain-containing_protein | 1 |
| DAUD_RS10515 | 2184650..2185237 | pyruvate/ketoisovalerate_oxidoreductase_subunit_gamma | 1 |
| DAUD_RS10550 | 2191444..2194155 | preprotein_translocase_subunit_SecA | 1 |
| DAUD_RS10660 | 2221392..2223221 | FAD-dependent_oxidoreductase | 1 |
| DAUD_RS11755 | 2226316..2229303 | S8_family_serine_peptidase | 1 |
| DAUD_RS10760 | 2238804..2240918 | copper_amine_oxidase_N-terminal_domain-containing_protein | 1 |
| DAUD_RS10920 | 2268355..2269020 | transposase | 1 |
| DAUD_RS11180 | 2318259..2319617 | peptidoglycan_DD-metalloendopeptidase_family_protein | 1 |
| DAUD_RS11230 | 2328349..2328576 | 30S_ribosomal_protein_S18 | 1 |


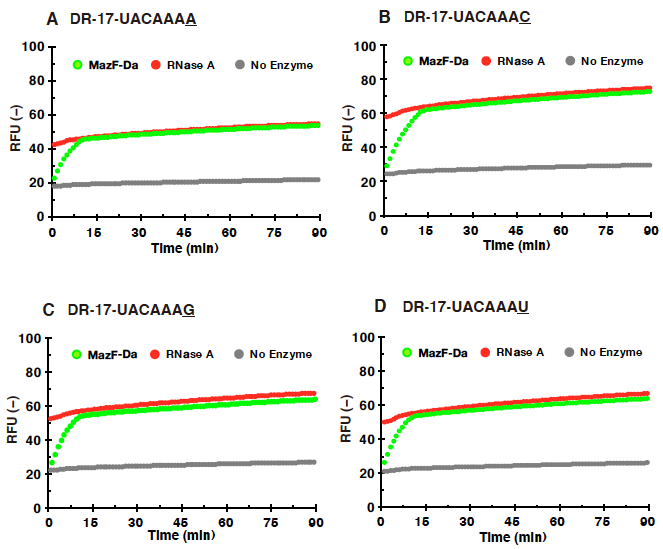


**Supplementary Figure S1. Investigation of the seven-base-specific ribonuclease MazF-Da**. MazF-Da (light-green) was reacted with each (A)–(D) fluorescent probe at 60 °C for 90 min; (A) DR-17-UACAAAA, (B) DR-17-UACAAAG, (C) DR-17-UACAAAC, and (D) DR-17-UACAAAU. Fluorescence intensities in the presence of RNase A (red) and in the absence of the enzymes (gray) at each time point were measured as control reactions.


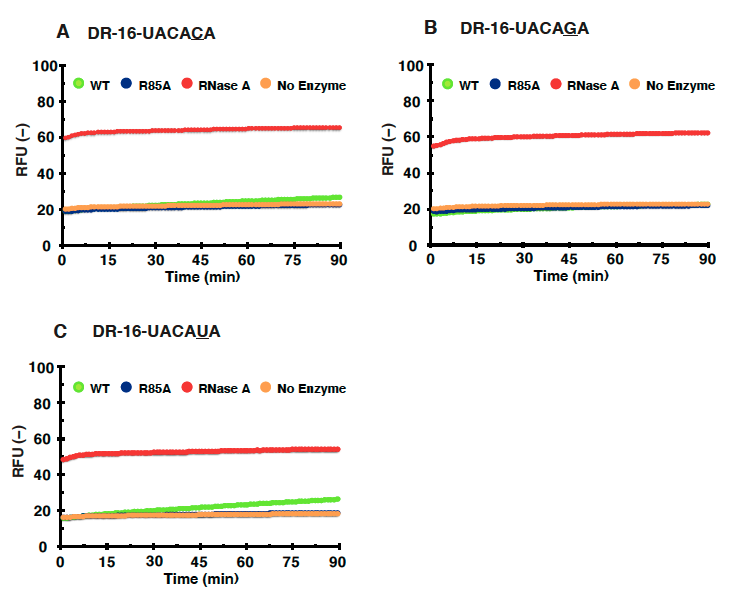


**Supplementary Figure S2. Identification of the cleavage sequence of the MazF-Da mutants.** The wild-type MazF-Da (light-green) and R85A mutant MazF-Da (navy) were reacted with each (A)–(C) fluorescent probe at 60 °C for 90 min; (A) DR-16-UACACA, (B) DR-16-UACAGA, and (C) DR-16-UACAUA. Fluorescence intensities in the presence of RNase A (red) and in the absence of the enzymes (orange) at each time point were measured as the control reactions.


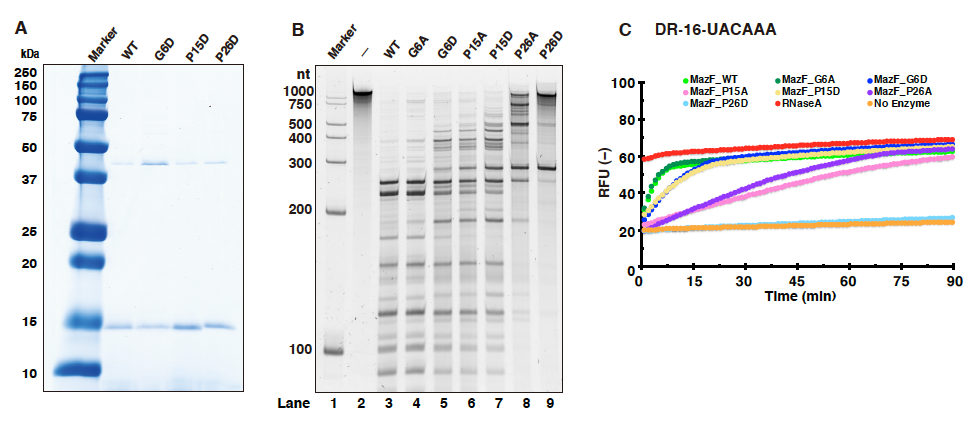


**Supplementary Figure S3. Comparison of enzymatic activities between alanine-substituted and aspartate-substituted mutants of MazF-Da.** (A) Molecular weight and purity of MazF-Da mutants expressed using the cell-free protein synthesis system were analyzed via SDS-PAGE and visualized using CBB staining. (B) Each MazF-Da mutant was reacted with substrate RNA for 90 min at 60 °C. The resultant digested RNA fragments were analyzed using urea gel. Lane 1, marker; lane 2, negative control without enzyme; lanes 3–9, wild-type, or each mutant (G6A, G6D, P15A, P15D, P26A, or P26D) of MazF-Da was added to substrate RNA. (C) Cleavage of the UACAAA sequence by MazF-Da mutants were measured continuously.
